# Correlation of biomarkers for parasite burden and immune activation with acute kidney injury in severe falciparum malaria

**DOI:** 10.1186/1475-2875-13-91

**Published:** 2014-03-12

**Authors:** Katherine Plewes, Annick A Royakkers, Josh Hanson, Md Mahtab Uddin Hasan, Shamsul Alam, Aniruddha Ghose, Richard J Maude, Pauline M Stassen, Prakaykaew Charunwatthana, Sue J Lee, Gareth DH Turner, Arjen M Dondorp, Marcus J Schultz

**Affiliations:** 1 Mahidol Oxford Tropical Medicine Research Unit, Faculty of Tropical Medicine, Mahidol University, Bangkok, Thailand; 2 Department of Anesthesiology, Zaans Medical Hospitals, Zaandam, the Netherlands; 3 Department of Intensive Care Medicine & Laboratory for Experimental Intensive Care and Anesthesiology (L E I C A), Academic Medical Center, University of Amsterdam, Amsterdam, the Netherlands; 4 Menzies School of Health Research, Darwin, Australia; 5 Chittagong Medical College Hospital, Chittagong, Bangladesh; 6 Centre for Tropical Medicine, Nuffield Department of Medicine, University of Oxford, Oxford, UK

**Keywords:** Acute kidney injury, Pathophysiology, Falciparum malaria, Soluble urokinase-type plasminogen activator receptor, Histidine rich protein-2

## Abstract

**Background:**

Acute kidney injury (AKI) complicating severe *Plasmodium falciparum* malaria occurs in up to 40% of adult patients. The case fatality rate reaches 75% in the absence of renal replacement therapy (RRT). The precise pathophysiology of AKI in falciparum malaria remains unclear. Histopathology shows acute tubular necrosis with localization of host monocytes and parasitized red blood cells in the microvasculature. This study explored the relationship of plasma soluble urokinase-type plasminogen activator receptor (suPAR), as a proxy-measure of mononuclear cell activation, and plasma *P. falciparum* histidine rich protein 2 (*Pf*HRP2*),* as a measure of sequestered parasite burden, with AKI in severe malaria.

**Methods:**

Admission plasma suPAR and *Pf*HRP2 concentrations were assessed in Bangladeshi adults with severe falciparum malaria (n = 137). Patients were stratified according to AKI severity based on admission creatinine clearance.

**Results:**

A total of 106 (77%) patients had AKI; 32 (23%), 42 (31%) and 32 (23%) were classified into ‘mild, ‘moderate’ and ‘severe’ AKI groups, respectively. Plasma suPAR and *Pf*HRP2 concentrations increased with AKI severity (test-for-trend *P* <0.0001) and correlated with other markers of renal dysfunction. Admission plasma suPAR and *Pf*HRP2 concentrations were higher in patients who later required RRT (*P* <0.0001 and *P* = 0.0004, respectively). In a multivariate analysis, both increasing suPAR and *Pf*HRP2 were independently associated with increasing urine neutrophil gelatinase-associated lipocalin concentration, a marker of acute tubular necrosis (β = 16.54 (95% CI 6.36-26.71) and β = 0.07 (0.02-0.11), respectively).

**Conclusions:**

Both sequestered parasite burden and immune activation contribute to the pathogenesis of AKI in severe falciparum malaria.

## Background

Acute kidney injury (AKI) complicating severe falciparum malaria is an independent predictor of death in both children and adults; without renal replacement therapy (RRT) the adult case fatality rate reaches 75% [1-3]. The incidence of AKI in severe malaria patients varies across studies, but approaches 40% in adults and 10% in children under five years old [4]. Both pre-renal (hypovolemia) and intrinsic renal factors can contribute; two thirds of adults present with anuria or oliguria [2,4]. Histopathology shows acute tubular necrosis (ATN) hypothesized to be caused by the observed sequestration of parasitized red blood cells (PRBCs) and accumulation of mononuclear cells in both glomerular and peritubular capillaries [5-7]. However, the relative contribution of each of these mechanisms cannot be assessed from *post-mortem* studies.

*Plasmodium falciparum* histidine-rich protein 2 (*Pf*HRP2) is released in distinct amounts at the moment of schizont rupture and is quantitatively related to parasite density [8,9]. The plasma concentration of *Pf*HRP2 estimates total body *P. falciparum* burden, including the sequestered biomass that causes obstructed microcirculatory flow in vital organs. Unlike the peripheral parasitaemia level, plasma *Pf*HRP2 concentration correlates strongly with disease severity and outcome [10-12].

Plasma soluble urokinase–type plasminogen activator receptor (suPAR) reflects the systemic level of cell surface urokinase-type plasminogen activator receptor (uPAR) expression, and has been proposed as a marker of immune activation via modulation of mononuclear cell adhesion and migration [13-15]. Cell-bound uPAR is expressed on several cell types including activated lymphocytes, monocytes, neutrophils, macrophages, vascular endothelial cells, and kidney podocytes [14,15]. In African children with acute malaria, plasma suPAR concentrations are elevated and associated with higher mortality [16,17]. suPAR has not been assessed previously in adults with severe falciparum malaria.

Neutrophil gelatinase-associated lipocalin (NGAL) is secreted predominantly in damaged distal tubular renal epithelial cells prior to neutrophil activation, and is an early marker of renal tubular cell damage [18-20]. NGAL concentration is more reliable as a measure of kidney injury than creatinine as it is less dependent on pre-renal factors [18-20]. Upregulation of NGAL in renal tubule cells may be induced by local release of cytokines from monocytes in the microcirculation after ischemic injury [19].

The relationship between plasma suPAR, as a proxy-measure of mononuclear cell immune activation, and plasma *Pf*HRP2, as a measure of sequestered parasite burden, with AKI in adult patients with falciparum malaria was assessed in this study. Severity of kidney dysfunction and damage was measured quantitatively by creatinine clearance and urine NGAL (a marker of acute tubular necrosis), respectively. Since kidney failure in severe malaria is a consequence of severe ATN, severe kidney tubular damage was assessed qualitatively by RRT requirement.

## Methods

### Study design and patients

The study was an analysis of a subset of patients enrolled in two clinical trials assessing N-acetylcysteine (2003–2005) and levamisole (2006–2010) as adjuvant therapy in severe falciparum malaria [21,22]. Of the 141 severe malaria patients enrolled in these two studies, all patients who had suPAR levels measured were included in this analysis (N = 137 out of 141). Both studies were conducted at Chittagong Medical College Hospital, Bangladesh, a 1,000-bed tertiary referral hospital with limited facilities for intensive care and RRT. Malaria transmission in this area is seasonal with peak transmission from June to August. This analysis builds on work presented from this cohort in 2011 [23]. Informed consent was obtained from each patient or designated family member. The Ministry of Health in Bangladesh, and Oxford Tropical Research Ethics Committee (OXTREC) granted ethical approval for both studies (registration numbers: ISRCTN20156397 and ISRCTN27232551).

Adults (≥16 years) with slide-confirmed severe *P. falciparum* malaria were recruited. Criteria for severe malaria included: coma (Glasgow Coma Score <11), shock (systolic blood pressure (SBP) <80 mmHg with cool extremities), severe anaemia (haematocrit <20% plus parasitaemia >100,000/μl), severe jaundice (total bilirubin >3.0 mg/dL plus parasitaemia >100,000/μl), hyperparasitaemia (peripheral asexual stage parasitaemia >10%), acidosis (venous bicarbonate <15 mmol/L), hyperlactataemia (venous lactate >4 mmol/L), hypoglycaemia (blood glucose <40 mg/dL), convulsions (≥ two in 24 hours), pulmonary oedema, and/or renal failure (serum creatinine >3 mg/dL).

Patients were treated with parenteral artesunate (Guilin No 2 Pharmaceuticals, China) and managed according to WHO treatment guidelines [24]. Supportive treatment, including fluid resuscitation, was provided according to the treating physician’s clinical judgment. RRT with haemodialysis or peritoneal dialysis was not available for all patients due to limited resources. Additional treatments have been previously described [21,22].

### Study procedures

On enrolment, a complete medical history and examination were performed, and venous blood and urine collected. Admission venous sodium, potassium, chloride, glucose, blood urea nitrogen, haemoglobin, haematocrit, pH and bicarbonate were assessed using a portable, handheld analyzer (iSTAT, Abbott, Illinois, USA). Peripheral parasitaemia was assessed on admission and every six hours until parasite clearance, defined as two consecutive negative blood films. Plasma, serum and urine samples were processed and stored at -80°C for further analysis in Bangkok, Thailand and Amsterdam, the Netherlands. The time and indication for RRT was recorded.

### Biomarker analysis

Plasma suPAR concentrations were measured using suPARnostic™ ELISA (ViroGates, Copenhagen, Denmark), according to the manufacturer’s instructions. Specimens were diluted to read within the calibration curve defined by quantitative standards. Reported results are the mean suPAR concentration (ng/ml) of duplicate wells for each specimen. Urine NGAL concentrations were measured using Human Lipocalin-2/NGAL ELISA (R&D Systems, Abingdon, UK) according to the manufacturer’s instructions. Multiple dilutions were tested in duplicate. The final urine NGAL concentration (pg/ml) was normalized to urinary creatinine and expressed as pg/mg of creatinine (uNGAL/Ucr). Plasma *Pf*HRP2 was assessed by commercial sandwich ELISA (Celisa, Cellabs, Sydney, Australia), according to the manufacturer’s instructions with minor modifications [9]. Pooled reference plasma from 20 subjects with parasitaemia >200,0000/μl was calibrated to purified *Pf*HRP2 standard curves (kindly provided by D Sullivan, John Hopkins School of Public Health, Baltimore, USA). Concentrations in duplicate plasma dilutions (1/25 to 1/3,125 in PBS/0.01%Tween) were determined according to the linear component of the standard curve. Cases where duplicates differed by more than 50% were re-assayed.

### Acute kidney injury

AKI was defined by the estimated creatinine clearance, which is an accepted surrogate of kidney function measured by the glomerular filtration rate (GFR). Severity of AKI was classified as mild, moderate or severe dysfunction based on admission creatinine clearance (CrCl): mild (CrCl = 60–89.9 ml/min), moderate (CrCl = 30–59.9 ml/min) and severe (CrCl <30 ml/min). No AKI was defined as CrCl ≥90 ml/min. CrCl was calculated using the Cockcroft-Gault formula [25]. No patient included in the analysis had a history of renal disease. Urine NGAL normalised to urine creatinine was used in this analysis as a biomarker for kidney damage, specifically acute tubular necrosis.

### Indication for renal replacement therapy

Three clinicians (JH, PC and AMD) blinded to suPAR and *Pf*HRP2 results, independently reviewed each patient file to determine whether patients met pre-defined criteria for RRT: hyperkalaemia (K >5.5 mmol/L), acidosis (pH <7.35, venous bicarbonate <15 mmol/L), fluid overload refractory to diuretics, uncontrolled seizures, pericarditis or rapid deterioration of renal function [23]. RRT was only deemed necessary in the setting of concomitant renal impairment (BUN >30 mmol/L or creatinine >200 μmol/L), as adults with severe malaria may have the above complications in the absence of AKI [23].

### Statistical analysis

Differences between AKI groups (mild, moderate, severe or no AKI) were compared by Student’s *t*-test and Mann–Whitney-*U* test for normally and non-normally distributed variables, respectively. Data were transformed to achieve a normal distribution where possible. A non-parametric test-for-trend, which is an extension of the Wilcoxon rank-sum test, was used to identify increasing or decreasing associations with AKI severity. Correlations between variables were assessed using Pearson’s correlation coefficient. A robust regression model was constructed to assess the contributions of suPAR, *Pf*HRP2, suPAR x *Pf*HRP2 interaction, age and SBP to the variation in uNGAL/Ucr. Age and SBP were included in the model as they are well-established risk factors for kidney injury. There were no significant interactions between CrCl and suPAR, nor CrCl and *Pf*HRP2, therefore these terms were not included in the models. To assess the relationship of the same independent variables with the indication for RRT, a logistic model with stepwise exclusion of non-significant covariates was used. Improved model fit with the inclusion of the interaction term was confirmed using the likelihood ratio test. A *P* value of less than 0.05 was considered significant. Statistical software used were STATA/IC 12.0 (STATA, TX, USA), and Prism 6 for Mac OS X (Graphpad Software, CA, USA).

## Results

**Figure 1:**
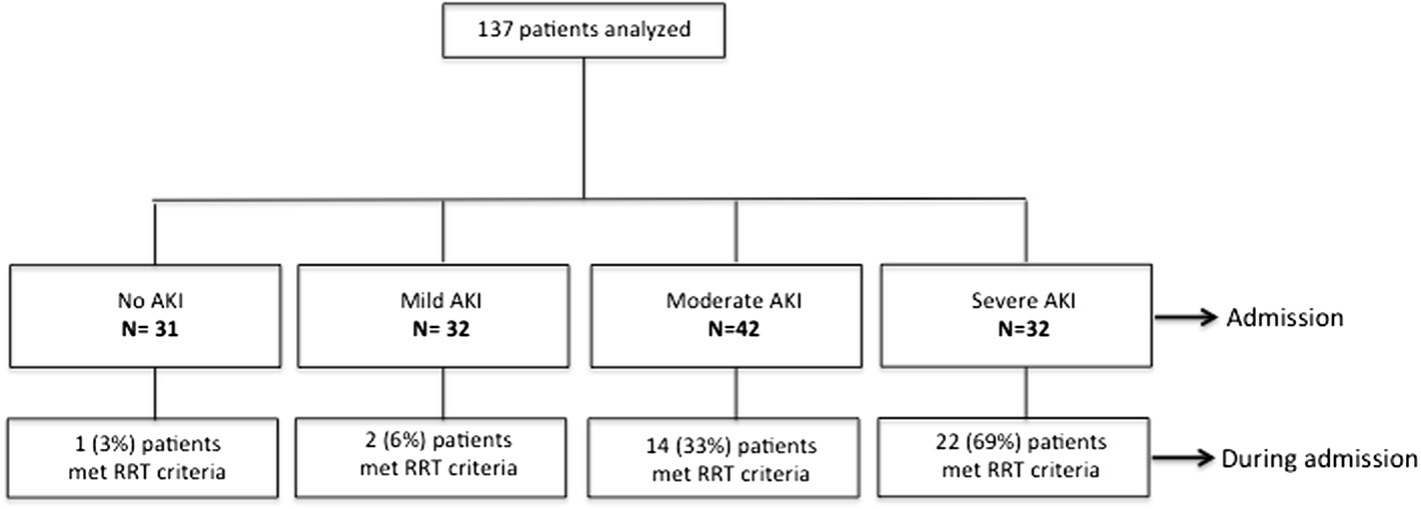
**Consort diagram.** After enrolment to the studies, patients admitted to Chittagong Medical College Hospital had blood and urine samples collected. Plasma and urine biomarkers were measured and correlated with the renal diagnosis and the subsequent hospital course. One patient with no AKI diagnosis on admission developed progressive renal impairment despite conservative measures and required RRT; this patient presented in deep coma with hyperlactataemia. AKI = acute kidney injury; RRT = renal replacement therapy.

One-hundred and thirty-seven adults with severe falciparum malaria were included in this analysis (Figure 1).

### Baseline characteristics

Baseline characteristics and patient outcomes are shown in Table 1. AKI was present in 106 patients (77%), of whom 32 (23%), 42 (31%) and 32 (23%) were classified as having mild, moderate and severe AKI, respectively. AKI classification using the WHO definition (creatinine >3 mg/dL) [24] rather than estimated CrCl, failed to identify 11 (34%) patients in the severe AKI group. These 11 patients had a geometric mean CrCl of 25 ml/min, and 5/11 (45%) required RRT. The WHO definition of AKI failed to identify 41/42 (98%) patients with moderate AKI (geometric mean CrCl 42 ml/min), of those 13/41 (32%) required RRT. There was unanimous consensus among three independent physicians on 39 patients (28%) who should have received RRT; 20 (51%) did not receive RRT for logistical reasons. Two patients received haemodialysis and 17 patients received peritoneal dialysis within 48 hours of admission. The most frequent indication for RRT was acidosis (Table 2). Of the 19 patients who received RRT, eight (42%) patients died; of the 20 patients who met criteria for RRT but could not receive it, 15 patients died (75%). The overall case fatality rate was 53/137 (39%); 50/53 (94%) had cerebral malaria, 33/53 (62%) had moderate or severe AKI, 35/53 (66%) had hyperlactataemia, and 33/53 (62%) had severe acidosis.

**Table 1:** Baseline characteristics by acute kidney injury severity and clinical outcome

**Variable**	**Total**	**No AKI**	**Mild AKI**	**Moderate AKI**	**Severe AKI**	**Test-for-trend**
	**(n = 137)**	**(n = 31)**	**(n = 32)**	**(n = 42)**	**(n = 32)**	
Age (years)^a^	35 (23–45)	26 (18–35)	33 (25–40)	35 (21–50)	40 (28–48)	**0.003**
Males^c^	110 (80)	27 (87)	27 (84)	30 (71)	26 (81)	0.301
**Clinical**						
SBP (mmHg)	111 (19)	116 (15)	113 (20)	108 (20)	107 (21)	**0.048**
RR (breaths/min)^b^	31 (30–33)	29 (27–30)	34 (31–37)	32 (29–34)	32 (28–35)	0.239
GCS (#/15)^a^	8 (6–11)	8 (7–10)	8 (5–10)	9 (6–11)	9 (5–12)	0.937
**Laboratory**						
HCT (%)	30 (8)	34 (7)	30 (9)	29 (8)	29 (8)	**0.011**
Parasitaemia	73209	46723	33560	174140	78018	0.070
(#/μL)^b^	(50362–106419)	(19688–110884)	(11984–93981)	(120635–251377)	(36814–165340)	
Creatinine	1.53	0.69	1.11	1.75	3.80	**<0.001**
(mg/dL)^b^	(1.37–1.72)	(0.63–0.76)	(1.03–1.21)	(1.60–1.91)	(3.26–4.43)	
BUN (mg/dL)^b^	42 (37–48)	21 (18–25)	27 (23–32)	57 (50–65)	88 (73–105)	**<0.001**
CrCl	50.6	123.3	72.5	41.7	19.3	**<0.001**
(ml/min)^b^	(44.9–57.0)	(112.8–134.8)	(69.8–75.3)	(39.0–44.5)	(17.0–21.8)	
Potassium (mmol/L)	4.4 (1.0)	4.0 (0.7)	4.4 (1.1)	4.4 (0.7)	4.6 (1.4)	**0.031**
Sodium (mmol/L)	133 (7)	129 (7)	134 (5)	133 (8)	133 (6)	0.069
Albumin (g/L)	29 (5)	31 (5)	30 (4)	28 (5)	27 (6)	**<0.001**
Total bilirubin (mg/dL)^b^	4.5 (3.8–5.4)	4.1 (2.8–5.9)	3.1 (2.3–4.1)	4.9 (3.5–6.7)	6.7 (4.7–9.6)	**0.011**
Direct bilirubin (mg/dL)^b^	1.8 (1.5–2.2)	1.2 (0.8–2.0)	1.1 (0.8–1.5)	2.2 (1.5–3.2)	3.4 (2.2–5.2)	**<0.001**
ALT (U/L)^b^	30 (25–36)	22 (17–28)	30 (21–43)	36 (25–51)	34 (23–51)	0.064
Base excess (mmol/L)	-9 (7)	-4 (4)	-5 (4)	-11 (7)	-13 (7)	**<0.001**
Bicarbonate (mmol/L)	17 (5)	21 (4)	19 (4)	14 (5)	14 (4)	**<0.001**
pH	7.37 (0.13)	7.42 (0.08)	7.42 (0.09)	7.35 (0.12)	7.30 (0.17)	**<0.001**
pCO2 (mmHg)	28 (7)	32 (6)	29 (7)	25 (8)	26 (7)	**<0.001**
Lactate (mmol/L)^b^	5.1 (4.6–5.6)	4.3 (3.7–5.1)	3.9 (3.2–4.7)	7.1 (6.0–8.3)	4.9 (3.8–6.4)	**0.029**
**Biomarkers**						
HRP2	1649	678	1413	2419	2833	**<0.001**
(ng/ml)^b^	(1297–2096)	(397–1156)	(967–2064)	(1715–3413)	(1648–4872)	
uNGAL/Ucr	1262	750	1,012	1281	2549	**<0.001**
(pg/mg cr)^b^	(1097–1451)	(589–956)	(745–1376)	(1066–1541)	(1943–3345)	
**Outcomes**						
RRT indicated^c^	39 (28)	1 (3)	2 (6)	14 (33)	22 (69)	**<0.001**
Death^c^	53 (39)	12 (39)	8 (25)	19 (45)	14 (44)	0.333

All values are mean (SD) unless otherwise specified; ^a^median (IQR), ^b^geometric mean (95% CI), ^c^number (%). *P* <0.05 using test-for-trend; significant in bold.

AKI = acute kidney injury; SBP = systolic blood pressure; RR = respiratory rate; GCS = Glasgow Coma Scale; HCT = haematocrit; BUN = blood urea nitrogen; CrCl = creatinine clearance; ALT = alanine transaminase; pCO2 = venous partial pressure carbon dioxide; HRP2 = *Plasmodium falciparum* histidine rich protein 2; suPAR = soluble urokinase-type plasminogen activator receptor; uNGAL/Ucr = urine neutrophil gelatinase-associated lipocalin corrected for urine creatinine; RRT = renal replacement therapy.

**Table 2:** Indication for renal replacement therapy

**Indication**	**Number**
Renal impairment and acidosis	20
Renal impairment and hyperkalaemia	0
Renal impairment, acidosis and hyperkalaemia	6
Renal impairment, acidosis and convulsions	2
Renal impairment, acidosis, hyperkalaemia and convulsions	3
Worsening renal function despite conservative measures	5
Worsening renal function and convulsions	1
Renal impairment and pulmonary oedema	1
Renal impairment, acidosis and pulmonary oedema	1
**Total**	**39**

Increasing severity of AKI correlated with increasing age, and decreasing SBP and haematocrit (test-for-trend *p* = 0.003, 0.048 and 0.011, respectively). Direct and indirect bilirubin increased and all measures of acid–base status (lactate, base deficit, bicarbonate, pH, pCO2) worsened with increasing kidney injury severity (test-for-trend *p* <0.001).

### Plasma soluble urokinase-type plasminogen activator receptor, *Plasmodium falciparum* histidine rich protein-2 and acute kidney injury

**Figure 2:**
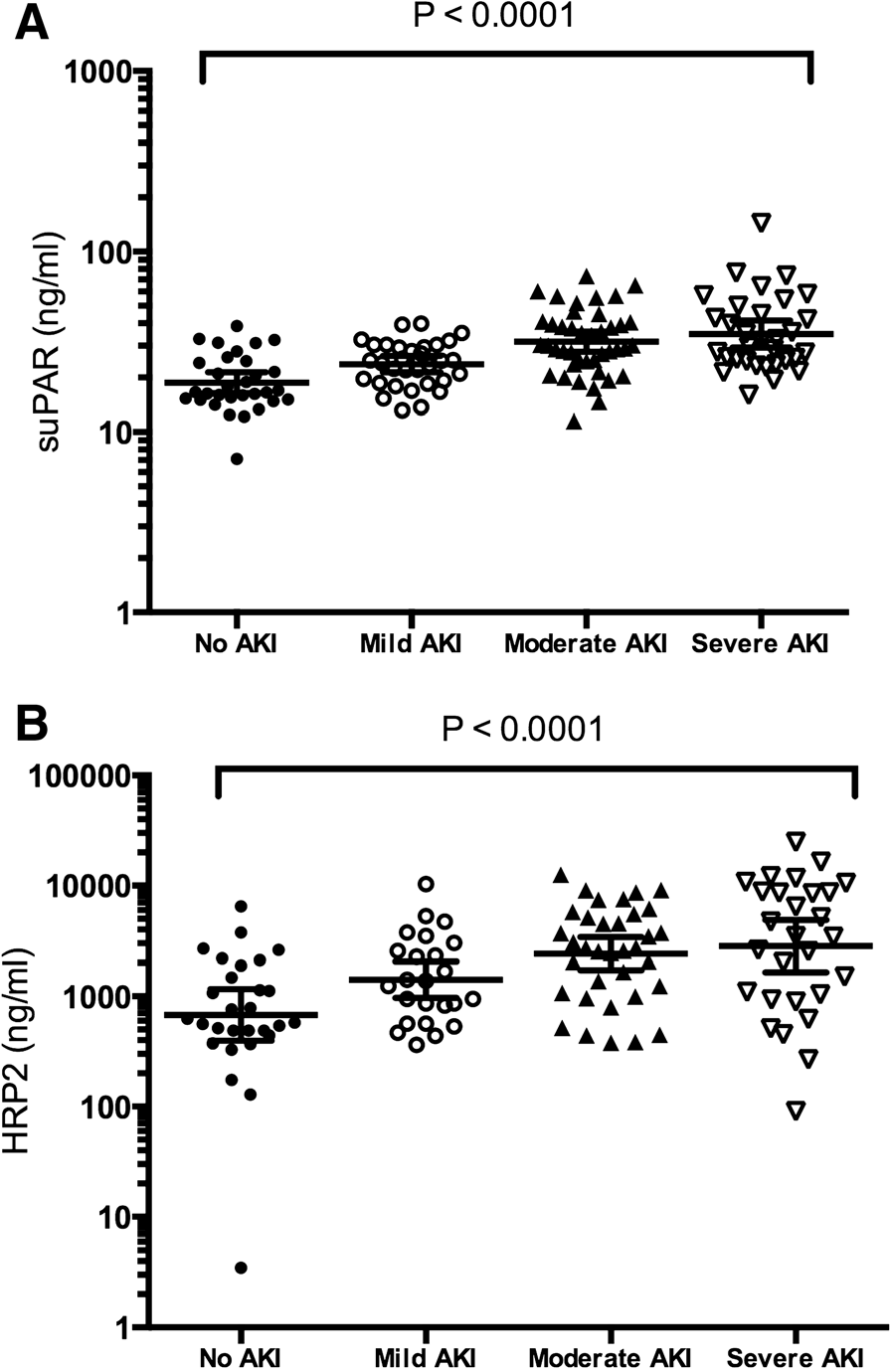
**Scatterplots of cohort stratified by admission acute kidney injury severity. (A)** Plasma soluble urokinase–type plasminogen activator receptor (suPAR), and **(B)** plasma HRP2 concentrations. Concentrations for both suPAR and HRP2 increased with increasing severity of AKI (test-for-trend *P* <0.0001). Geometric mean and 95% confidence intervals displayed. AKI = acute kidney injury; HRP2 = *P. falciparum* histidine rich protein 2.

**Figure 3:**
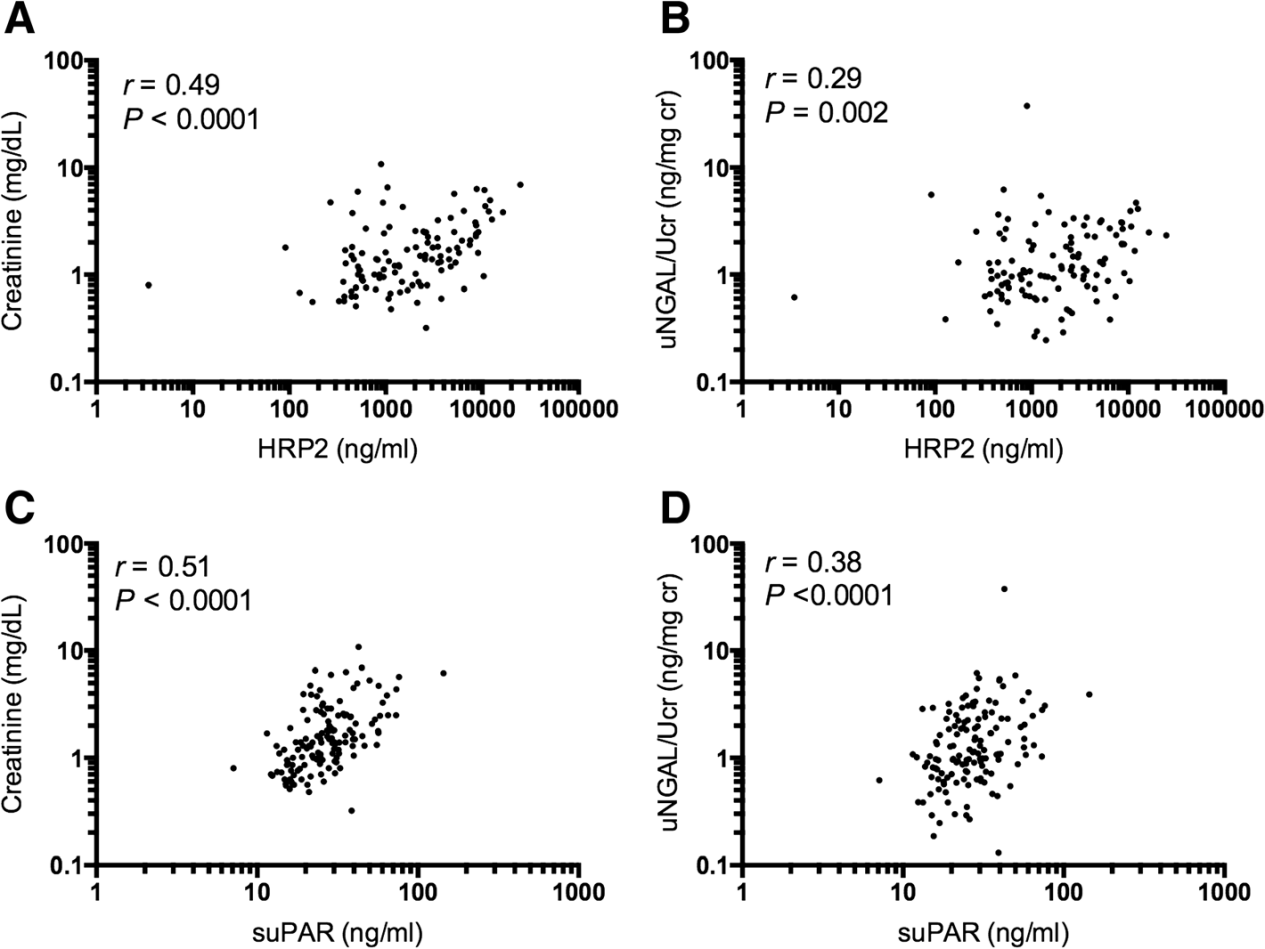
**Correlations of variables involved in acute kidney injury.** Correlations of variables proposed to be involved in the pathogenesis of acute tubular necrosis in malaria associated AKI with marker of renal dysfunction (creatinine) and marker of kidney damage (uNGAL/Ucr). Pearson’s correlation coefficient between plasma *P. falciparum* histidine rich protein 2 (HRP2) and **(A)** serum creatinine; and **(B)** uNGAL/Ucr. Pearson’s correlation coefficient between suPAR and **(C)** serum creatinine; and **(D)** uNGAL/Ucr. All variables were log transformed (log_10_) for correlation plots and Pearson’s analysis. suPAR = soluble urokinase–type plasminogen activator receptor; uNGAL/Ucr = urine neutrophil gelatinase-associated lipocalin corrected for urine creatinine.

Unlike peripheral parasitaemia, admission plasma *Pf*HRP2 increased with worsening kidney function (test-for-trend *p* <0.001, Figure 2). Plasma *Pf*HRP2 increased with both increasing creatinine (*r* = 0.49, *P* <0.001) and increasing uNGAL/Ucr (*r* = 0.29, *P* <0.002) (Figure 3A-B). Peripheral parasitaemia had a borderline correlation with serum creatinine (*r* = 0.17, *P* = 0.05). Admission plasma suPAR increased with increasing AKI severity (test-for-trend *p* <0.001, Figure 2). There was a strong positive correlation between suPAR and both creatinine (*r* = 0.51, *P* <0.001) and uNGAL/Ucr (*r* = 0.38, *P* <0.001) (Figure 3C-D).

Plasma suPAR strongly correlated with *Pf*HRP2 (*r* = 0.54, *P* <0.001); the interaction term (suPAR × *Pf*HRP2) also increased with both increasing creatinine (*r* = 0.58, *P* <0.0001), increasing uNGAL/Ucr (*r* = 0.38, *P* <0.0001) and AKI severity (test-for-trend *p* <0.0001). This was further evaluated using linear regression with uNGAL/Ucr as the dependent variable, representing acute tubular necrosis. In the univariate analysis suPAR, *Pf*HRP2 and suPAR x *Pf*HRP2 were significantly associated with a higher concentration of uNGAL/Ucr (Table 3). Multivariate analysis showed that only plasma suPAR and *Pf*HRP2 were independently associated with higher uNGAL, predicting 18% of the variation in uNGAL/Ucr concentrations (*R*^*2*^ = 0.18; Table 3).

**Table 3:** Linear regression analysis of variables contributing to urine NGAL/Ucr variability in patients with severe malaria

	**Univariate analysis**		**Multivariate model (*R***^***2***^ **= 0.18)**	
**Variable**	**β (95% CI)**^**a**^	***P***	**β (95% CI)**^**a**^	***P***
suPAR	21.57 (12.20-30.95)	**<0.001**	16.54 (6.36-26.71)	**0.002**
*Pf*HRP2	0.11 (0.06-0.15)	**<0.001**	0.07 (0.02-0.11)	**0.003**
suPAR x *Pf*HRP2	0.002 (0.001-0.003)	**<0.001**	---	**---**
SBP	0.16 (-8.62-8.94)	0.971	---	---
Age	-12.02 (-25.32-1.28)	0.076	---	**---**

^a^Regression coefficient (β) with 95% confidence intervals (CIs) showing the estimated increase in the urine concentration of NGAL (pg/ml) corrected for urine creatinine (uNGAL/Ucr pg/mg of creatinine) predicted by 1 unit increases in the independent (predictor) variables using robust regression analysis. Significant in bold.

**Figure 4:**
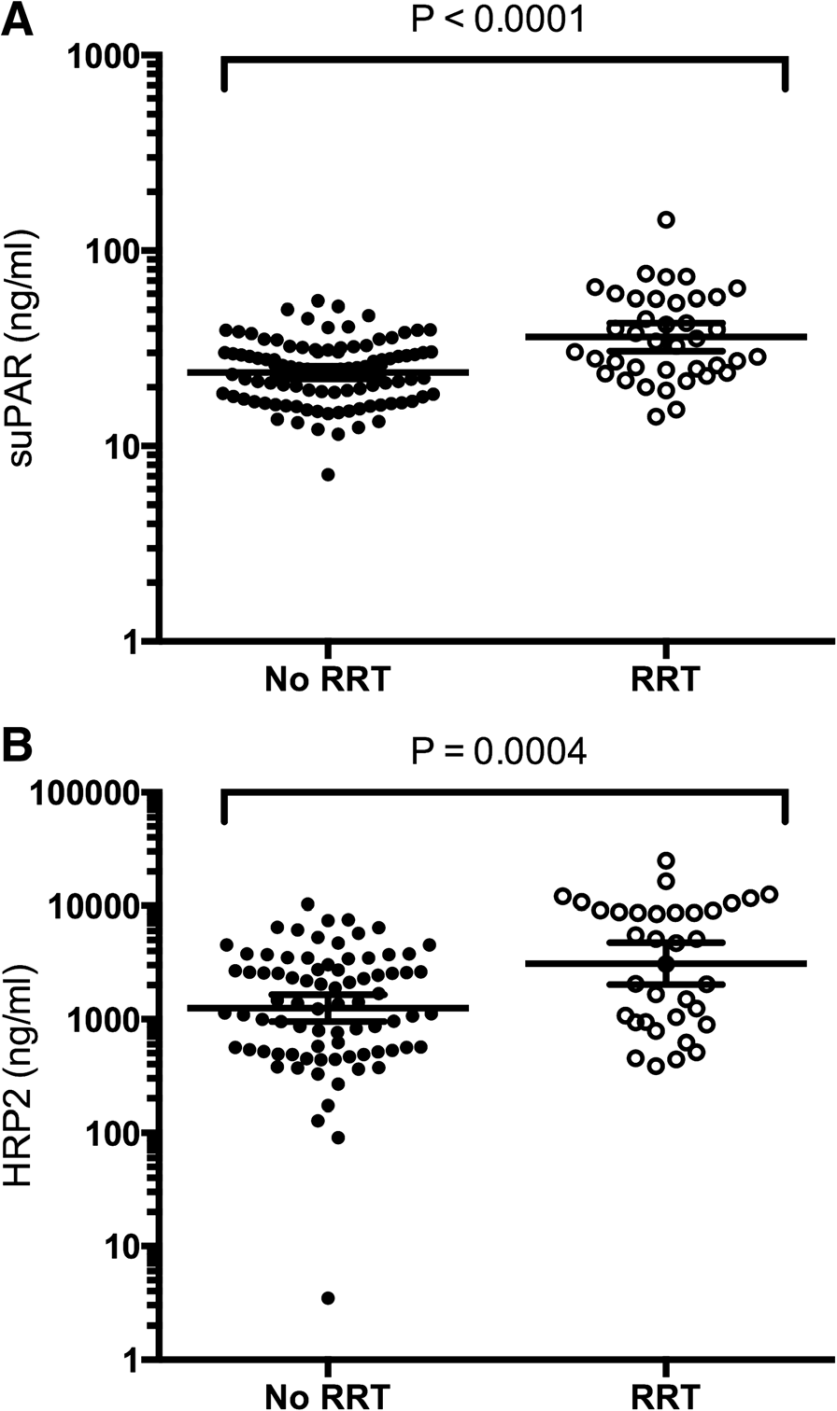
**Scatterplots of cohort stratified by renal replacement therapy requirement. (A)** Plasma suPAR, and **(B)** plasma HRP2. Biomarkers were log transformed (log_10_) for normality. *T-*tests were performed for comparisons between groups. RRT = renal replacement therapy; suPAR = soluble urokinase–type plasminogen activator receptor; HRP2 = *P. falciparum* histidine rich protein 2.

Plasma suPAR and *Pf*HRP2 concentrations were higher in patients requiring RRT (*P* <0.001, Figure 4). Logistic regression was performed to further evaluate this relationship with RRT as the binary dependant variable, representing severe tubular damage in severe malaria. In univariate logistic analysis, increased suPAR, *Pf*HRP2 and suPAR x *Pf*HRP2 were associated with increased odds for RRT requirement (Table 4). In a stepwise, multivariate, logistic regression model, increasing plasma suPAR, *Pf*HRP2 and age increased the odds of RRT requirement (*R*^*2*^ = 0.27; Table 4).

**Table 4:** Logistic regression analysis of variables predicting renal replacement therapy in patients with severe malaria

	**Univariate analysis**		**Multivariate model (*R***^***2***^ **= 0.27)**	
**Variable**	**OR (95% CI)**^**b**^	***P***	**OR (95% CI)**^**b**^	***P***
suPAR	1.07 (1.04-1.11)	**<0.001**	1.07 (1.03-1.12)	**0.002**
*Pf*HRP2^1/2^	1.03 (1.02-1.05)	**<0.001**	1.02 (1.00-1.04)	**0.026**
suPAR^1/2^ x *Pf*HRP2^1/2^	1.01 (1.00-1.01)	**<0.001**	---	**---**
SBP	1.01 (0.99-1.03)	0.522	---	---
Age	1.02 (0.99-1.05)	0.123	1.05 (1.01-1.10)	**0.010**

^b^Odds ratios (OR) with 95% CIs were determined for renal replacement therapy (RRT) associated with 1-U changes in the independent variables. Significant variables are shown in bold. *Pf*HRP2 was square root transformed to normalize skewed data. Significant in bold.

OR = odds ratio; CI = confidence intervals; uNGAL/Ucr = urine neutrophil-associated gelatinase lipocalin corrected for urine creatinine; suPAR = soluble urokinase plasminogen activator receptor; *Pf*HRP2 = *P. falciparum* histidine rich protein 2; SBP = systolic blood pressure.

### Relationship between plasma soluble urokinase-type plasminogen activator receptor, *Plasmodium falciparum* histidine rich protein-2 and mortality

Plasma suPAR and *Pf*HRP2 concentrations were not significantly higher in patients who died compared with those who survived: 29.03 (25.38-33.22) versus 25.57 (23.27-28.10) ng/ml for suPAR (*P =* 0.12) and 1,685 (1,162-2,444) versus 1,629 (1,185-2,237) for *Pf*HRP2 (*P* = 0.89). Plasma suPAR and *Pf*HRP2 were significantly elevated in patients who were acidotic (base deficit >3.3 mmol/L) compared to those who were not: 29.63 (27.07-32.44) versus 20.67 (18.02-23.71) ng/ml for suPAR (*P* <0.001) and 2,025 (1,569-2,613) versus 816 (469–1,420) for *Pf*HRP2 (*P* = 0.001). Standard base deficit was significantly elevated in patients who died compared to survivors; mean (SD), 11.8 (7.5) versus 6.5 (5.1) (*P* <0.001).

## Discussion

In this study of adult patients with severe falciparum malaria, admission plasma suPAR and *Pf*HRP2 were strongly and independently associated with AKI, and a later requirement for RRT. As suPAR is an indirect measure of mononuclear cell activation, and *Pf*HRP2 a measure of sequestered parasite biomass, these results suggest that both mechanisms contribute to the pathogenesis of AKI. These findings are concordant with *post-mortem* renal histopathological findings in patients with falciparum malaria that shows both PRBC sequestration and mononuclear cell infiltration in glomerular and peritubular capillaries [6].

Multivariate regression analysis showed that plasma *Pf*HRP2 independently correlated with uNGAL, and a requirement for RRT. Histopathology of falciparum malaria-associated AKI shows a significant increase in sequestration among patients with a creatinine >3 mg/dL, which defines AKI according to WHO malaria treatment guidelines [6]. Overall, disease severity and the severity of metabolic acidosis have also been shown to correlate strongly with plasma *Pf*HRP2 concentrations [9-12,26]. *Pf*HRP2 was significantly elevated in patients who were acidotic. Peripheral parasitaemia does not reflect the pathogenic-sequestered parasite burden that causes impaired microvascular flow, and the correlation between parasitaemia and total parasite burden is variable. This might explain why the presented results contrast with other studies, in that an association between hyperparasitaemia and AKI severity was not found [6,27,28]. However, plasma *Pf*HRP2, representing the total parasite biomass including the sequestered parasites, strongly correlated with AKI defined by either WHO or CrCl classification. The correlation between *Pf*HRP2 and creatinine was also stronger than the correlation between peripheral parasitaemia and creatinine. Sequestered PRBCs occluding the renal microcirculation may explain why increasing the overall renal blood flow in severe malaria does not result in a change in renal oxygen consumption [29]; as only patent vessels free of sequestered PRBCs would continue to be perfused and PRBC occluded vessels would not be recruited.

Plasma suPAR increased with deteriorating renal function defined by CrCl classification and WHO criteria, urine NGAL and independently predicted RRT requirement. Plasma suPAR is positively correlated with creatinine in patients with Hantavirus and critically ill patients, and inversely correlated with estimated glomerular filtration rate (GFR) in chronic kidney disease [30-33]. Histology of brain tissue from cerebral malaria patients shows an increase in uPAR expression in macrophage and endothelial cells spatially limited to areas with PRBCs sequestered in the microvasculature [34]. This is the first time elevated suPAR has been shown to be associated with kidney injury and dysfunction in malaria. This supports that the histopathological mononuclear cell accumulation in glomerular and peritubular capillaries contribute to the acute tubular necrosis in patients with a creatinine above and below 3 mg/dL, despite a difference in observed sequestration of PRBCs. It is possible that sequestration acts as an endothelial nidus and subsequent positive feedback loop for increased suPAR-mediated mononuclear cell recruitment and immune activation resulting in more severe tubular damage. Cell-to-cell contact between endothelial cells and monocytes increases suPAR release [14] and suPAR subsequently recruits more monocytes via its chemo-attractant effector function [35,36]. Upon schizont rupture, haemozoin (malaria pigment) acts as antigen to activate monocytes resulting in release of pro-inflammatory cytokines and chemokines [37,38]. This could initiate a positive feedback loop since TNF-α increases expression of uPAR on monocytes resulting in accelerated cleavage of suPAR and consequent monocyte recruitment [35,38]. Indeed, mononuclear cells loaded with pigment are observed in glomerular and peritubular microvessels on histopathology [6]. As elevated suPAR concentrations have not been shown to be directly pro-inflammatory [39], the accumulation of activated monocytes releasing cytokines are the likely effectors contributing to renal tubular cell damage in severe malaria.

In this study there was no association between suPAR and mortality, which contrasts with a study of African children with malaria where increased suPAR concentrations were associated with poor clinical outcomes [16]. This may be because there was a low mortality rate (2%) in the African study, suggesting less severe disease. The entire cohort presented here was severely ill with a high mortality rate and higher suPAR concentrations compared to the African study [16]; including uncomplicated malaria patients in this analysis may have shown an association between suPAR concentration and mortality.

There are limitations to this study. Both suPAR and HRP2 measured in the plasma are not organ specific markers of monocyte activation and sequestration, respectively. However, kidney specific markers of these phenomena currently do not exist. The justification of the analyses of plasma suPAR and HRP2 is based on the histopathology that has shown sequestration of PRBCs and mononuclear cell infiltration in the kidney. Since suPAR was measured in plasma, the concentrations measured likely represent production by activated host immune cells, in particular mononuclear cells, however endothelial sources could also contribute. As suPAR has renal clearance, elevated plasma levels can be a consequence of reduced GFR [31,33]. However, in the multiple regression models, interaction terms of CrCl, suPAR and *Pf*HRP2 did not reveal a significant contribution, and the association between suPAR and indication for RRT was also found in patients presenting without AKI on admission. It has been shown that plasma HRP2 half life is not correlated with biochemical evidence of kidney dysfunction [9]. Urine NGAL as a biomarker for renal tubular damage has limitations, as its excretion depends on the timing in relation to the initial renal injury [40]. However, NGAL is an ‘induced’ biomarker, thus excretion is continued with ongoing renal stress [40]. Using renal biopsy as a reference standard for ATN was not feasible or ethically indicated in this study, so RRT requirement was used as an alternative endpoint. Finally, the sample size in this study was relatively small and patient data were reviewed retrospectively. However, this assessment was done according to pre-defined criteria and three independent specialist physicians were in full agreement.

## Conclusions

This study suggests that parasite sequestration and immune activation contribute to the pathogenesis of AKI in severe falciparum malaria. Early diagnosis of falciparum malaria and treatment with artemisinin-based anti-malarials will rapidly eliminate ring-staged parasites, preventing their microvascular sequestration in vital organs, including the kidney. Deeper understanding of the immunopathological mechanisms underlying AKI in severe malaria might reveal novel, targeted, treatment options to further prevent this common and life-threatening complication.

## Abbreviations

suPAR: Plasma soluble urokinase-type plasminogen activator receptor; PfHRP2: Plasma *Plasmodium falciparum* histidine rich protein 2; AKI: Acute kidney injury; RRT: Renal replacement therapy; uNGAL: Urine neutrophil gelatinase-associated lipocalin; Ucr: Urine creatinine; uNGAL/Ucr: Urine neutrophil gelatinase-associated lipocalin corrected for urine creatinine; CrCl: Creatinine clearance; ATN: Acute tubular necrosis; SBP: Systolic blood pressure; PRBCs: Parasitized red blood cells; GFR: Glomerular filtration rate; WHO: World Health Organization.

## Competing interests

MJS is an advisor of Virogates A/S, Denmark. He has no financial interests in the company. All other authors declare that they have no competing interests.

## Authors’ contributions

KP contributed to managing patients, sample collection, statistical analysis and manuscript preparation. AAR contributed to data analysis and manuscript preparation. JH contributed to patient management, sample collection and manuscript revision. MUH, SA and AG supervised the study and clinical care of the patients. RJM and PC contributed to patient management, data collection and entry and manuscript revision. PMS provided the suPAR measurements. SJL assisted with statistical analysis. GDHT assisted with data interpretation and critical manuscript revision. AMD and MSJ were the senior supervisors, conceived of and designed the study, assisted with statistical analysis and manuscript revision. All authors read and approved the final manuscript.
